# Supplementary material for: Enhanced intermolecular coulombic decay due to sulfur heteroatoms in thiophene dimer
Source: Commun Chem. 2025 May 15;8:151. doi: 10.1038/s42004-025-01547-6 (PMC12081764; doi:10.1038/s42004-025-01547-6)
Supplement: Supplementary file 2 — Supplementary material [file 42004_2025_1547_MOESM2_ESM.pdf]

Supplementary material: Enhanced intermolecular Coulombic decay due to sulfur heteroatoms in thiophene dimer

Deepthy Maria Mootheril[1], Anna D Skitnevskaya[2], Xueguang Ren[3], Mevlut Dogan[1], Alexander I Kuleff[4], Alexander B Trofimov[2], Lorenz S Cederbaum[4], Thomas Pfeifer[1], and Alexander Dorn[1]

[1]*Max-Planck-Institut für Kernphysik, Saupfercheckweg 1, 69117 Heidelberg, Germany*
[2]*Federal Research Center, A. E. Favorsky Irkutsk Institute of Chemistry of the Siberian Branch of the Russian Academy of Sciences, Favorskogo str. 1, 664033 Irkutsk, Russia*
[3]*MOE Key Laboratory for Nonequilibrium Synthesis and Modulation of Condensed Matter, School of Physics, Xi'an Jiaotong University, 710049 Xi'an, China*
[4]*Theoretische Chemie, Physikalisch-Chemisches Institut, Universität Heidelberg, 69120 Heidelberg, Germany*

I Filtering Coulomb explosions events of dimers from the background

The time-of-flight coincidence map for thiophene is shown in figure S1. It depicts the coincidence between two thiophene ions. The vertical and horizontal lines (within black dashed rectangles) are the false coincidences of different mass fragments with singly charged thiophene monomer ions with small momentum. The diagonal line (within the red dashed rectangle) shows the Coulomb explosion (CE) of two intact thiophene ions from a dimer, whereas the background is produced by other CE events giving rise to either two thiophene ions or its fragments. Some of such processes are the following:

1. CE of a dimer which also involves a loss of one or more neutral hydrogen
2. Two thiophene ions originating from a larger cluster
3. Two thiophene fragments which involve hydrogen losses and originate from a larger cluster

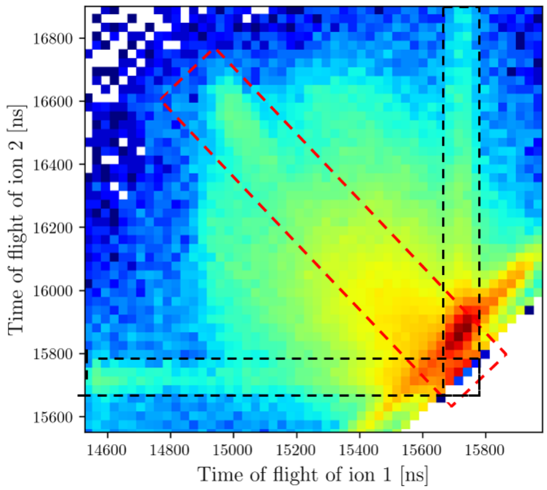

Figure S1: Time-of-flight coincidence map for thiophene including the CE of dimers and the background events.

To filter the desired CE events of the dimer from the background events we use two techniques:

i Take events with the sum of the times-of-flight of the first ion and second ion to be within a small time window of ∼100 ns, around two times the time-of-flight of the

thiophene parent ion. Due to the equal momentum gain in opposite directions, the events from the CE of the dimer lie on a diagonal. The sum of the times-of-flight of these events is a constant which is equal to two times the sum of time-of-flight of parent ion. This removes false coincidences and some of the background around the diagonal. However, the false coincidences and some background within the red dashed rectangle remain after this procedure.

ii Select events with vector sum of ion momenta close to zero. Since the ions gain equal momentum from the Coulomb repulsion and undergo back-to-back emission, the vector sum of the ion momenta should be equal to zero. Considering also the momentum spread due to the limited resolution we expect the event from the CE to have a momentum sum below 20 au. To select the event we plot a 2D histogram with the magnitude of the vector sum of ion momenta versus the kinetic energy release (KER) as shown in the figure S2a. The separate island which lies below 20 au and around 2.6 eV KER gives the Coulomb explosion event from the dimers. The corresponding KER distribution for the CE events is shown in figure S2b.

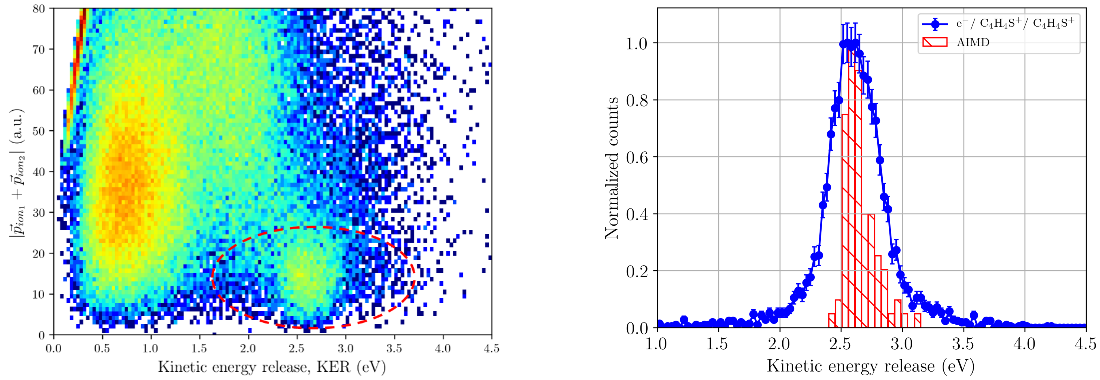

(a) (b)

Figure S2: **Filtering of CE events from background.** (a) Sum of the momenta of the ions versus the kinetic energy release (KER). The small island within the red dashed circle shows the CE events from the dimers. (b) KER spectrum after filtering the background.

Figures S3 show the momentum coincidences in X, Y and Z directions corresponding to the events within the marked island in figure S2a after the filtering procedure. The width of the diagonal line in the Y and Z directions is due to the limited resolution in those directions.

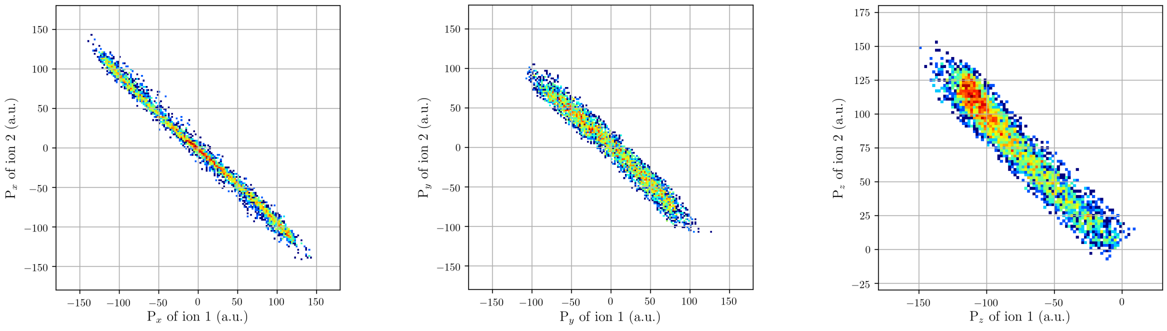

(a) (b) (c)

Figure S3: **Momentum coincidence plots after background filtering.** (a) P_x of ion 2 versus P_x of ion 1. (b) P_y of ion 2 versus P_y of ion 1. (c) P_z of ion 2 versus P_z of ion 1.

II Procedure for determination of energy shift to ADC calculations

II.1 ADC(3) for single ionization

The typical error of IP-ADC(3) in the outer-valence region is below 0.5 eV (see, e.g., [1]). This is, however, for states with mainly 1h ('one-hole') character that dominate in the outer-valence region of the IP spectra and are treated in the framework of the IP-ADC(3) approach at the third order of perturbation theory. In this work we consider electronic decays of cationic states in the inner-valence region in which the one-electron picture of ionization is known to break down and the states have a large 2h-1p ('two-holes-one-particle') contribution. Within the IP-ADC(3) method, the 2h-1p states are treated through first order, resulting in, sometimes, substantially larger errors. Consequently, the 2h-1p states can appear considerably shifted and with incorrect intensity, but, due to their large number, the overall spectral shape in this energy region is usually good. In this work, we evaluate the shift by comparing theoretical and experimental photoelectron spectra of thiophene monomer as shown in figure S4. Two intense bands in the inner-valence region are compared with those of the experiment and an average energy shift of 1.2 eV is determined. Thus, the same shift was applied to the inner-valence region of the IP spectrum of the thiophene dimer.

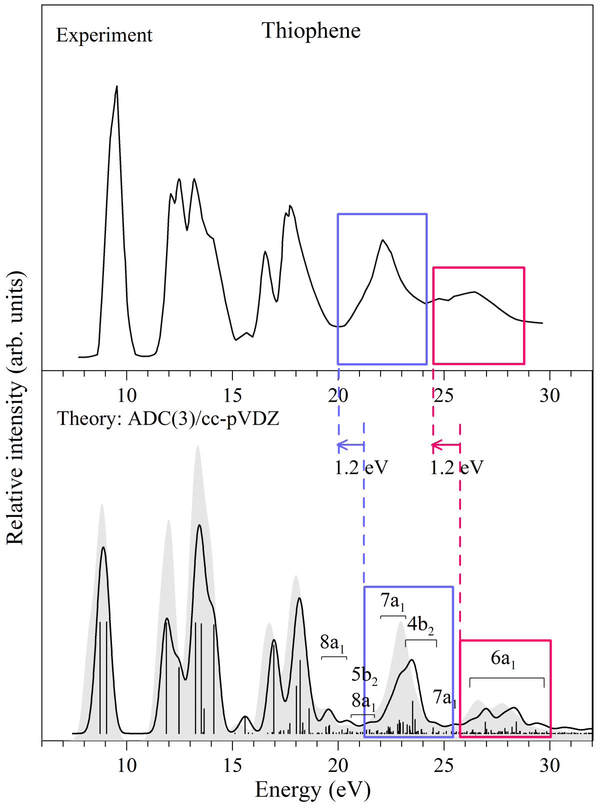

Figure S4: **Estimation of energy shift for single ionization spectrum.** Upper panel: experimental ionization spectrum of thiophene. Lower panel: theoretical spectra of thiophene monomer (black curve) and T-shaped thiophene dimer (gray shaded curve). The data for thiophene monomer is adopted from [2], the single ionization spectrum is recalculated here with the cc-pVDZ basis set.

II.2 ADC(2) for double ionization

Spectra of doubly ionized states are calculated at the ADC(2)/cc-pVDZ level of theory. The second-order ADC scheme for double ionization is known to have red shifts of energies compared to experiment [3], while the structure of the spectra is usually reproduced quite well [4]. Table S1 shows an

example of calculated DIP values for the thiophene monomer from the current and a previous work [4]. The corresponding experimental energies for the same orbitals are given. It can be seen that the shift is not the same for different transitions. Based on the comparison with the experimental data for the monomer and similarly to Ref. [4], the DIP spectrum of the thiophene dimer was blue shifted by 1 eV.

Table S1: **Estimation of energy shift for double ionization spectrum.** Calculated energies of the dicationic states using ADC(2) theory and their comparison with the experimental results for determining the energy shift.

	DIP-ADC(2) values without shift		
	cc-pVDZ	cc-pVTZ [4]	Experiment [4]
$^3B_2(1a_2^{-1}2b_1^{-1})$	23.3	23.6	
$^1B_2(1a_2^{-1}2b_1^{-1})$	23.6	24.0	
$^1A_1(2b_1^{-1})$	25.1	25.4	26.1
$^1B_1(2b_1^{-1}6a_2^{-1})$	27.3	27.5	29.1

Table S1 shows that the DIP blue shift can be chosen differently depending on the choice of basis set and the state in comparison with the experimental data in [4]. Figure S5 presents the double ionization spectra with varying blue shifts (0.7 eV, 1.0 eV, and 1.8 eV). Notably, the overlap between the band produced by the inner-valence ionization in the 20–24 eV range and the energy gap between the ICD and Auger thresholds remains largely unchanged. This suggests that our interpretation that these inner-valence ionized states contribute to the enhancement of the low-energy ICD electrons remains valid regardless of the chosen blue shift for the DIP spectra.

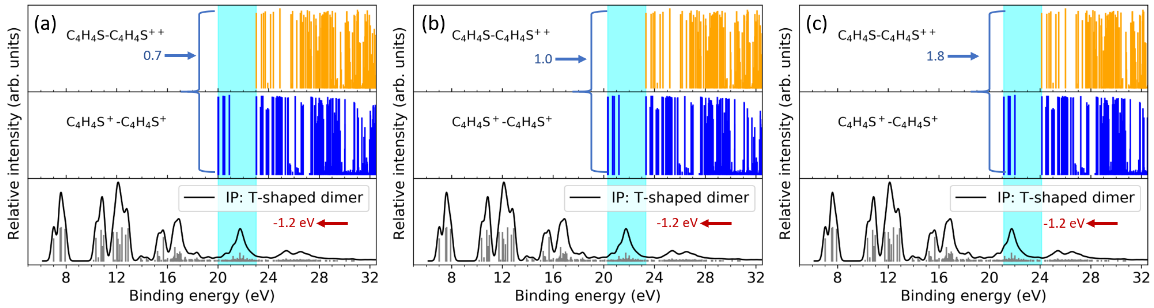

Figure S5: **Analysis of energy blue shift for the double ionization spectrum.** The top and middle panels of (a), (b), and (c) show the double ionization spectra for the T-shaped thiophene dimer with delocalized and localized charges, respectively, at energy blue shifts of 0.7 eV, 1.0 eV, and 1.8 eV. The lower panel of all three figures presents the single ionization spectra of the T-shaped thiophene dimer with a red shift of 1.2 eV.

III Comparison of electronic structure calculations for thiophene monomer, T-shaped dimer, and sandwich dimer

Figure 6 shows a comparison of the single and double ionization spectra of sandwich and T-shape thiophene dimers, together with those of the monomer. The corresponding dimer structures are also shown. One can see that in the IP spectrum (bottom panel in figure S6c) the intensities of the 1h states in the outer-valence region (upto ∼20 eV) differ to some extent in shape and intensity for the different conformers and the monomer but appear at very similar energies. However, in the inner-valence region (>20 eV), where the states are subject to electronic decays, the shape and amplitudes of the spectra of the conformers are similar. Thus, no significant dependence of the decay probabilities and energies on the orientation of the thiophene molecules in the dimer is expected.

Likewise, the DIPs (top panel in figure S6c) for the T-shaped and sandwich conformers are very similar. This will give rise to a similar enhancement in the low-energy region for both dimers. One can expect minor changes in the ICD spectrum, with a very similar overall shape.

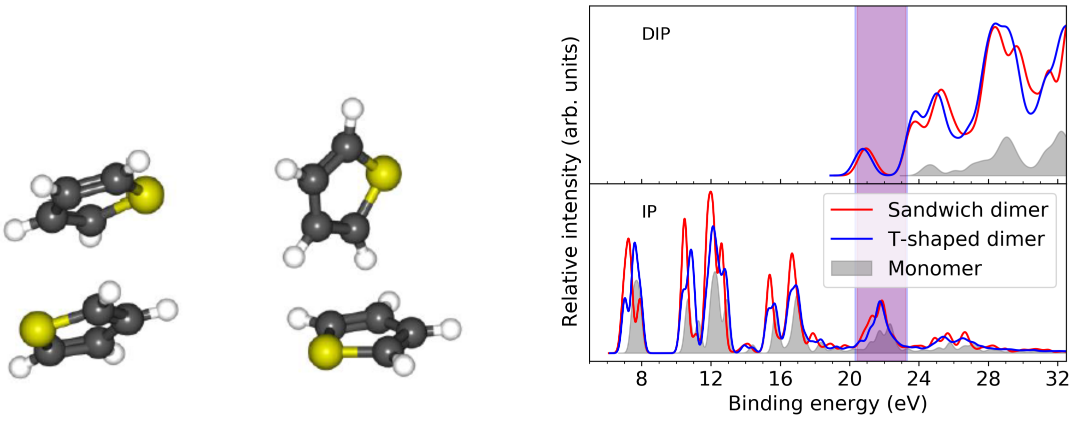

(a) (b) (c)

Figure S6: **Comparison between different conformers of thiophene dimer.** Structures of (a) sandwich dimer and (b) T-shaped dimer of thiophene. (c) Single ionization (IP) (bottom panel) and double ionization (DIP) (top panel) spectra of sandwich and T-shaped dimer and monomer of thiophene. DIP spectra of dimers include both localized (Auger/ETMD) and delocalized (ICD) states.

IV Estimation of sequential ionization events

The partial cross section for the ICD channel can be estimated from the partial cross sections for the production of C_3H^+ and $C_2H_2^+$ ions. Here an assumption is made that, while in the dimer the ionization of the inner-valence orbital $7a_1$ leads to ICD and in the monomer the molecule dissociates to C_3H^+ and $C_2H_2^+$ ions. Therefore, the sum of partial cross-sections for the C_3H^+ and $C_2H_2^+$ dissociation channels give the partial cross-section for ICD process. The partial cross-sections for the dissociation channels can be obtained using the relative abundance for the production of C_3H^+ and $C_2H_2^+$ ions which are 1.49% and 0.68% respectively [5] and from the total ionization cross-section of thiophene at 68 eV which is given by $\sigma_{thio}^{total}(\mathrm{E_0} = 68\text{ eV}) = 12.2 \times 10^{-16}\text{ cm}^2$ [6]. This yields a cross-section value for the monomer ionization channels (dissociation channels) to $\sigma_{\mathrm{C_3H^+}}(\mathrm{E_0} = 68\text{ eV}) = 18.16 \times 10^{-18}\text{ cm}^2$ and $\sigma_{\mathrm{C_2H_2^+}}(\mathrm{E_0} = 68\text{ eV}) = 8.36 \times 10^{-18}\text{ cm}^2$. Assuming that the $7a_1^{-1}$ vacancy in the dimer decays via ICD, the partial cross-section σ_{ICD} is estimated to be $26.52 \times 10^{-18}\text{ cm}^2$.

The partial cross-section for sequential ionization (SI) can be estimated as the product of the partial ionization cross-section of outer-valence orbital by the incoming projectile and the probability of undergoing outer-valence ionization in a subsequent collision with the neighbouring molecule which is given as follows:

$$\sigma_{\mathrm{SI}} = \sigma_{\mathrm{C_4H_4S^+}}(\mathrm{E_0} = 68\text{ eV})\frac{\sigma_{\mathrm{C_4H_4S^+}}(\mathrm{E_0} = 59\text{ eV})}{4\pi R^2} \tag{1}$$

For the equilibrium intermolecular distance R = 5 Å [7], $\sigma_{\mathrm{SI}} = 4.12 \times 10^{-18}\text{ cm}^2$. Therefore, the SI events constitute 13.4% of the total CE events.

Supplementary references

[1] Adrian L. Dempwolff, Alexander C. Paul, Alexandra M. Belogolova, Alexander B. Trofimov, and Andreas Dreuw. Intermediate state representation approach to physical properties of molecular electron-detached states. II. Benchmarking. *The Journal of Chemical Physics*, 152(2):024125, 01 2020.

[2] A. B. Trofimov, I. L. Zaitseva, T. E. Moskovskaya, and N. M. Vitkovskaya. Theoretical investigation of photoelectron spectra of furan, pyrrole, thiophene, and selenole. *Chemistry of Heterocyclic Compounds*, 44(9):1101–1112, Sep 2008.

[3] L. Storchi, F. Tarantelli, S. Veronesi, P. Bolognesi, E. Fainelli, and L. Avaldi. The Auger spectroscopy of pyrimidine and halogen-substituted pyrimidines. *The Journal of Chemical Physics*, 129(15):154309, 10 2008.

[4] P. Linusson, L. Storchi, F. Heijkenskjöld, E. Andersson, M. Elshakre, B. Pfeifer, M. Colombet, J. H. D. Eland, L. Karlsson, J.-E. Rubensson, F. Tarantelli, and R. Feifel. Double photoionization of thiophene and bromine-substituted thiophenes. *The Journal of Chemical Physics*, 129(23):234303, 12 2008.

[5] Nist chemistry webbook, nist standard reference database 69, 1997.

[6] P. Mozejko, E. Ptasińska-Denga, and Cz Szmytkowski. Cross sections for electron collision with five-membered ring heterocycles. *European Physical Journal D*, 66(2), 2012.

[7] Seiji Tsuzuki, Kazumasa Honda, and Reiko Azumi. Model chemistry calculations of thiophene dimer interactions: Origin of π-stacking. *Journal of the American Chemical Society*, 124(41):12200–12209, 2002.
