# Supplementary material for: Enhanced intermolecular coulombic decay due to sulfur heteroatoms in thiophene dimer
Source: Commun Chem. 2025 May 15;8:151. doi: 10.1038/s42004-025-01547-6 (PMC12081764; doi:10.1038/s42004-025-01547-6)
Supplement: Supplementary file 3 — Description of additional supplementary files [file 42004_2025_1547_MOESM3_ESM.pdf]

Description of Additional Supplementary Files

File name: Supplementary Data 1

Description: Source data for figure 2 (a)-(d)

File name: Supplementary Data 2

Description: Source data for figure 3 (a)-(c)

File name: Supplementary Data 3

Description: Source data for figure 4

File name: Supplementary Data 4

Description: Source data for figure 5

File name: Supplementary Data 5

Description: Coordinates of heterocyclic dimers used for spectra calculations, in angstroms.
