# Supplementary material for: Enhanced intermolecular coulombic decay due to sulfur heteroatoms in thiophene dimer
Source: Commun Chem. 2025 May 15;8:151. doi: 10.1038/s42004-025-01547-6 (PMC12081764; doi:10.1038/s42004-025-01547-6)
Supplement: Supplementary file 8 — Supplementary data 5 [file 42004_2025_1547_MOESM8_ESM.docx]

Coordinates of heterocyclic dimers used for spectra calculations, in angstroms.

Thyophene dimer - T-shaped

6 2.297423000 1.287661000 0.715363000

6 2.291882000 -0.000196000 1.245733000

16 2.291197000 -1.199297000 -0.001619000

6 2.291027000 0.003010000 -1.245899000

6 2.296924000 1.289517000 -0.712240000

1 2.295374000 2.185469000 1.336762000

1 2.282686000 -0.303160000 2.292626000

1 2.280835000 -0.297294000 -2.293548000

1 2.294209000 2.188904000 -1.331350000

6 -2.964508000 -1.310707000 0.000472000

6 -3.539092000 -0.042419000 0.000031000

16 -2.331341000 1.197511000 -0.000316000

6 -1.046058000 0.039119000 0.000144000

6 -1.538068000 -1.262573000 0.000549000

1 -3.555170000 -2.229582000 0.000736000

1 -4.596439000 0.223559000 -0.000117000

1 -0.011758000 0.377889000 0.000078000

1 -0.884615000 -2.137684000 0.000862000

Thyophene dimer - PD

6 1.436887000 -1.567543000 0.230060000

6 2.262062000 -0.877805000 -0.653863000

16 2.396286000 0.792535000 -0.220291000

6 1.345424000 0.588726000 1.139476000

6 0.912452000 -0.728172000 1.257402000

1 1.222385000 -2.634204000 0.132796000

1 2.796261000 -1.259904000 -1.524111000

1 1.103035000 1.440439000 1.774328000

1 0.240370000 -1.062778000 2.049642000

6 -1.682702000 1.566400000 0.109942000

6 -2.413588000 0.573156000 0.755554000

16 -2.247653000 -0.953579000 -0.042531000

6 -1.194475000 -0.284518000 -1.241438000

6 -0.983910000 1.074876000 -1.033432000

1 -1.659092000 2.603127000 0.453211000

1 -3.039299000 0.658799000 1.644279000

1 -0.784968000 -0.921115000 -2.024900000

1 -0.349729000 1.681619000 -1.682302000

Benzen T-shaped dimer

1 0.000000000 0.000000000 -0.124867000

6 0.000000000 1.218233000 1.670990000

6 0.000000000 1.219884000 3.079256000

6 0.000000000 0.000000000 0.965643000

6 0.000000000 0.000000000 3.783343000

6 0.000000000 -1.218233000 1.670990000

6 0.000000000 -1.219884000 3.079256000

1 0.000000000 0.000000000 4.877665000

1 0.000000000 -2.164068000 1.119825000

1 0.000000000 2.167033000 3.627529000

1 0.000000000 -2.167033000 3.627529000

1 0.000000000 2.164068000 1.119825000

6 0.000000000 1.408489000 -2.375587000

6 1.219962000 0.704229000 -2.375455000

6 -1.219962000 0.704229000 -2.375455000

6 1.219962000 -0.704229000 -2.375455000

6 -1.219962000 -0.704229000 -2.375455000

6 0.000000000 -1.408489000 -2.375587000

1 2.167454000 -1.250880000 -2.370079000

1 -2.167454000 -1.250880000 -2.370079000

1 2.167454000 1.250880000 -2.370079000

1 -2.167454000 1.250880000 -2.370079000

1 0.000000000 -2.502555000 -2.373050000

1 0.000000000 2.502555000 -2.373050000

Furan PD dimer with C2h symmetry

6 -0.650179078 -1.836737453 -0.692514422

6 0.650179078 1.836737453 0.692514422

6 -0.650179078 -1.836737453 0.692514422

6 0.650179078 1.836737453 -0.692514422

6 0.650179078 -1.589683699 1.061688421

6 -0.650179078 1.589683699 -1.061688421

6 0.650179078 -1.589683699 -1.061688421

6 -0.650179078 1.589683699 1.061688421

8 1.451859326 -1.444129475 0.000000000

8 -1.451859326 1.444129475 0.000000000

1 -1.492762240 -1.993146373 -1.335304333

1 1.492762240 1.993146373 1.335304333

1 -1.492762240 -1.993146373 1.335304333

1 1.492762240 1.993146373 -1.335304333

1 1.143015555 -1.496673918 1.988187453

1 -1.143015555 1.496673918 -1.988187453

1 1.143015555 -1.496673918 -1.988187453

1 -1.143015555 1.496673918 1.988187453

Pyridine PD dimer

6 1.414729000 1.306591000 -0.745524000

6 2.205927000 1.044205000 0.385805000

6 2.442447000 -0.293233000 0.752103000

7 1.953842000 -1.356814000 0.072826000

6 1.195768000 -1.086938000 -1.014978000

6 0.900462000 0.213623000 -1.462117000

1 1.196418000 2.332282000 -1.055840000

1 2.632593000 1.857486000 0.979888000

1 3.056833000 -0.526642000 1.628277000

1 0.808844000 -1.956960000 -1.555634000

1 0.276767000 0.361190000 -2.347488000

6 -1.414946000 -1.306741000 0.745170000

6 -2.206087000 -1.043928000 -0.386108000

6 -2.442335000 0.293645000 -0.752072000

7 -1.953629000 1.356971000 -0.072455000

6 -1.195644000 1.086682000 1.015308000

6 -0.900514000 -0.214052000 1.462074000

1 -1.196855000 -2.332562000 1.055216000

1 -2.632875000 -1.856987000 -0.980407000

1 -3.056606000 0.527402000 -1.628233000

1 -0.808667000 1.956492000 1.556264000

1 -0.276789000 -0.361921000 2.347386000

Pyrrole dimer

7 1.437953000 -0.000220000 1.224136000

7 -0.908579000 0.000029000 -0.725668000

6 1.760948000 1.132023000 0.510240000

6 2.328037000 0.716310000 -0.700493000

6 2.328403000 -0.715786000 -0.700686000

6 1.761526000 -1.132112000 0.509938000

6 -1.602541000 -1.129559000 -0.355892000

6 -2.793531000 -0.714978000 0.252785000

6 -2.793549000 0.714799000 0.252922000

6 -1.602566000 1.129528000 -0.355667000

1 0.041708000 0.000073000 -1.091942000

1 0.930356000 -0.000460000 2.101312000

1 1.566590000 -2.125440000 0.907043000

1 2.713122000 -1.372369000 -1.479648000

1 2.712319000 1.373297000 -1.479331000

1 1.565259000 2.125143000 0.907491000

1 -1.206720000 2.122625000 -0.557618000

1 -3.571003000 1.371611000 0.641401000

1 -3.570965000 -1.371885000 0.641144000

1 -1.206657000 -2.122605000 -0.558020000

Imidazole dimer

7 3.626978000 -0.447488000 -0.624106000

6 3.643357000 0.331045000 0.518602000

6 2.327154000 -0.607511000 -0.907459000

6 2.343966000 0.638527000 0.921150000

7 1.520987000 0.030247000 -0.002692000

1 4.577380000 0.629921000 0.990834000

1 1.927215000 -1.169147000 -1.748832000

1 1.952164000 1.217681000 1.753989000

1 0.488157000 0.046082000 -0.008373000

7 -1.395748000 0.040242000 -0.001995000

6 -2.254221000 0.953383000 -0.587250000

6 -2.179494000 -0.881695000 0.565947000

6 -3.575425000 0.579172000 -0.370270000

7 -3.501048000 -0.588375000 0.363796000

1 -1.882754000 1.821040000 -1.127672000

1 -1.843078000 -1.755347000 1.118549000

1 -4.521896000 1.025090000 -0.663422000

1 -4.287394000 -1.135227000 0.695586000
